# The Emerging Roles and Therapeutic Potential of Extracellular Vesicles in Infertility

**DOI:** 10.3389/fendo.2021.758206

**Published:** 2021-10-22

**Authors:** Guannan Zhou, Yuanyuan Gu, Fangyue Zhou, Menglei Zhang, Ganrong Zhang, Ligang Wu, Keqin Hua, Jingxin Ding

**Affiliations:** ^1^ Department of Gynecology, The Obstetrics and Gynecology Hospital of Fudan University, Shanghai, China; ^2^ Department of Gynecology, Shanghai Key Laboratory of Female Reproductive Endocrine Related Diseases, Shanghai, China; ^3^ Changning Maternity and Infant Health Hospital, East China Normal University, Shanghai, China; ^4^ State Key Laboratory of Molecular Biology, CAS Center for Excellence in Molecular Cell Science, Shanghai Institute of Biochemistry and Cell Biology, Chinese Academy of Sciences, Shanghai, China

**Keywords:** extracellular vesicles, female infertility, male infertility, IVF, therapeutics

## Abstract

Infertility is becoming much more common and affects more couples. The past years witnessed the rapid development of the diagnosis and treatment upon infertility, which give numerous coupled more opportunities become parents. Extracellular vesicles are known as nano-sized membrane vesicles to play a major role in intracellular communication. In recent years, several basic and clinical studies have tried to investigate the correlation between the reproductive health/disorder and extracellular vesicles. However, the mechanism is still unclear. In this review, we reviewed the relationship between reproductive physiology and extracellular vesicles, and then collectively focused on the recent findings on the relationship between extracellular and infertility, and its consequent influence on the novel insight regarding the therapeutic strategies for infertility in the future clinical practice.

## Introduction

Infertility (subfertility) is defined as a disease characterized by the failure to establish a clinical pregnancy after 12 months of regular and unprotected sexual intercourse” (1, 2). Infertility is common and is estimated that affects 1/6 couples at reproductive-age worldwide (3, 4). Several key steps are involved in achieving pregnancy including follicular development, fertilization, implantation and so on. Thus, premature ovarian insufficiency (5), polycystic ovary syndrome (6), endometriosis (7), uterine fibroids and endometrial polyps may play vital roles in female infertility.

Extracellular vesicles are bubbles with lipid bilayer structure of 30-5000 nm in diameter that secreted by different cells (8, 9). It was widely acknowledged that extracellular vesicles are produced by the fusion of multivesicular membrane with the plasma membrane, contain diverse cargos including proteins (10), mRNAs and microRNAs (11). Therefore, extracellular vesicles could act as important mediators of cell-cell message communication and exchange of substance that involved in numerous physiological and pathological processes (12, 13). Numerous studies have clarified that extracellular vesicles participant in cancers (14), immune responses (15), pregnancy and so on. Increasing studies indicated that extracellular vesicles derived from diverse types of cells are involved in infertility (16). Furthermore, the therapeutic potential of extracellular vesicles in infertility have been increasingly addressed in this field. While the diameter of exosomes ranged from 50 to 150 nm, extracellular vesicles formed at the plasma membrane can be of this size range or larger (up to 5 mm). Different extracellular vesicles subtypes cannot be separated according to size or density. Extracellular vesicles with similar sized can be classified into several types of extracellular vesicles based on biogenesis, size and biophysical contents: exosomes (ranged from 50 to 150 nm) secreted upon fusion of multivesicular compartments with the plasma membrane, microvesicles (or ectosomes) (ranged from 100 to 1,000 nm) and apoptotic bodies (ranged from 100 to 5,000 nm) released directly from the plasma membrane, and exomeres (ranged from 30 to 50 nm). The establishment of a formal International Society of Extracellular Vesicles (ISEV) has defined standards for the experimental characterization of extracellular vesicles, and encouraged the use of ‘extracellular vesicle’ as a generic term for all secreted vesicles, and as a keyword in all publications. Despite there were difference among diverse types of extracellular vesicles including biogenetic mechanisms and contents, it is difficult to distinguish different vesicle types after they are released or secreted from a cell. Thus, the clear descriptive function from diverse extracellular vesicles is still unclear worth further exploring.

Although an evidence-based, cost-effective and safer fertility treatment developed in the recent years, several issues (including the physical and psychological pressure, the substantial financial burden of infertility treatment, the unsatisfied success rate and so on) are still unsolved. Better understanding the molecular mechanisms of disorders related to infertility, and further developing timely effective therapeutics are urgent issues in this field.

In this review, we summarized the existing research on extracellular vesicles in fertility biology and infertility disorder. We aimed to illustrate the relationship between the extracellular vesicles and infertility (referring to both the female infertility and the male infertility), and also considered priorities for future research. Moreover, we summarized the extracellular vesicles in in-vitro fertilization (IVF) and the applications of extracellular vesicles in treating infertility, which might be an invaluable tool for the intervention of infertility and other related infertility disorders.

## Fertility Physiology and Extracellular Vesicles

### Male Fertility Healthy Physiology and Extracellular Vesicles

It is widely acknowledged that spermatogenesis is a vital and complex process during the whole process of male fertility physiology (17–19). This process requires the collaboration of numerous genes, hormones, proper temperature combined with other environmental factors. While sperms isolated from the testicle are generally immotile and immature, the maturation of sperm during transit through the epididymis is important for acquiring capacity of gaining motility and fertilization. Several studies indicate that part of this process is correlated to extracellular vesicles in transferring RNAs, proteins, and other materials from the epididymis to sperm (20).

Extracellular vesicles derived from epididymis (epididymsomes (21)), ranged between 50 and 250 nm, play a vita role to sperm during epididymal transit. It was reported that epididymis-extracellular vesicles could transfer a variety of proteins to surrounding epithelial cells and sperm, and further regulate transcription/translation within these cells (22). In addition, it appears that epididymis-extracellular vesicles carrying microRNAs are transferred between epididymal epithelial cells and spermatozoa to regulating sperm maturation (23). What’s more, several studies depicted that epididymis-extracellular vesicles content affected by paternal metabolic contents would further influence the healthy of offspring (22).

Extracellular vesicles-associated proteins are involved in the biological processes such as cell growth and maintenance, metabolism (24). Also, human seminal extracellular vesicles contain diverse small non-coding RNAs that modulate female reproductive tract (25) to support embryo development (26).

**Figure 1:**
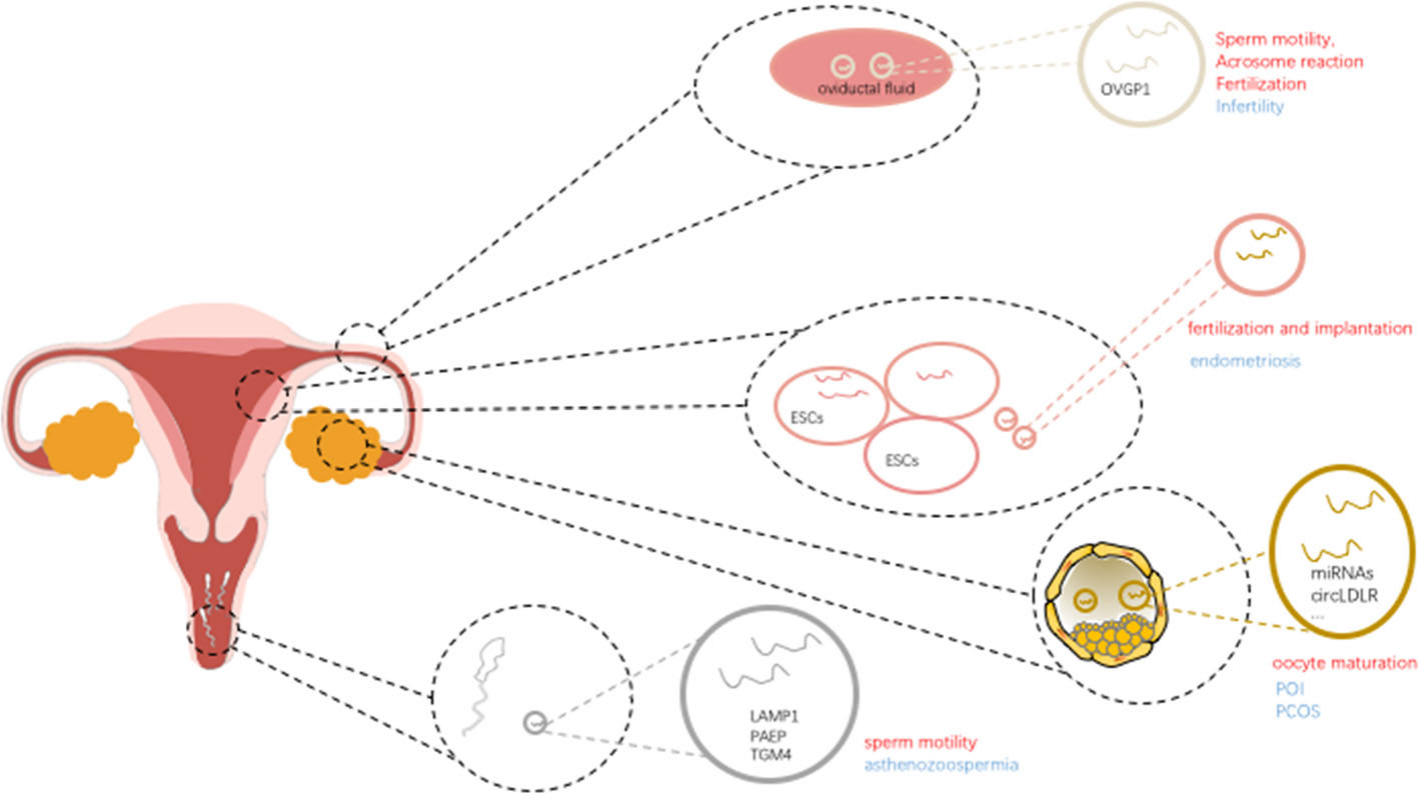
Schematic diagram showing the impact of extracellular vesicles in infertility.

In addition, extracellular vesicles derived from the vaginal, uterine, and fallopian tube fluid have been shown to bind sperm, and to prevent premature activation of the acrosome reaction in mice (27). Furthermore, these extracellular vesicles and encapsulated protein cargos (28) have also been found in the human female reproductive tract, suggested that extracellular vesicles involve in a highly conserved and important mechanism in supporting sperm (29) (**Figure 1**).

### Female Fertility Healthy Physiology and Extracellular Vesicles

When it comes to the female reproductive physiology, follicular development and maturation are regarded as complicated processes which involve intercellular communication between the maturing oocyte, cumulus cells and mural granulosa cells. The ovarian follicular development (including recruitment, selection and growth of follicles, followed by atresia or dominance, ovulation and formation of the corpus luteum and finally luteolysis) needs complicated coordination in the multi-steps duration. The role of communication (30) between theca cells, mural granulosa cells, cumulus cells as well as the oocyte in the ovary are critical for ovulation of a high-quality oocyte and further potential development into an embryo. It is quiet clear that the appropriate communication mediated by extracellular vesicles among diverse types of cells within the ovarian follicle is critical for the growth and maturation of healthy oocytes (31), particularly in fertilization and development into embryos.

It is clear that extracellular vesicles are present in ovarian follicular fluid, extracellular vesicles could mediate the delivery of molecular cargo (including proteins, microRNAs) between the different follicular cells to play a role in cell-to-cell communication in regulating follicle development and oocyte maturation. Extracellular vesicle miR-23a, regulates the apoptosis of human granulosa cells through affect the XIAP (which may contribute to the etiology of POF) and the caspase signaling cascade in human granulosa cells, was reported involved in the oocyte maturation (32). In addition, extracellular vesicle miR-21-5p (33) derived from follicular fluid plays a dynamic role in preimplantation embryo development by regulating apoptotic proteins by targeting PI3K/AKY and JAK/STAT3 signaling pathways in the process of cellular communication. These studies clearly depicted that extracellular vesicles involved in various aspects of follicular growth and maturation by transferring microRNAs. Further studies suggest that extracellular vesicles microRNAs play an important role in follicular development and cellular communication within the ovarian follicle by regulating critical signaling pathways, including TGF-β and WNT signaling. Based on the high-throughput sequencing results, extracellular vesicles miR-31-5p was found to promote the proliferation of GCs and progesterone synthesis *via* the WNT/β-actin pathway by targeting the SFRP4 follicle growth inhibitor and further regulating the physiological function of GCs, which is vital in follicle development (34, 35) (**Figure 1**).

In addition, fallopian tube plays a vital role in absorbing and transporting eggs, fertilization, and initial embryonic development. The contents including extracellular vesicles derived from the fallopian tube influence sperm motility, acrosome reaction, and fertilization. Extracellular vesicles associated miR-30d derived from the endometrial fluid was taken up by trophoblastic cells of murine embryos, and was involved in modifying the embryo transcriptome and its adhesive phenotype. Extracellular vesicles derived from oviductal fluid contain the OVGP1 (oviduct specific protein) and influence the sperm motility, acrosome reaction and fertilization (36). Also, when it comes to the proper communication and regulation between gametes/embryos and the fallopian tube, extracellular vesicles also paly important role in the multi-steps process. For example, proteins including endothelial nitric oxide synthase (eNOS), PMCA1 and PMCA4 can be delivered to sperm by extracellular vesicles *via* a fusogenic mechanism, and contributing to the sperm viability (37–39).

## Female Infertility and Extracellular Vesicles

### Endometriosis and Extracellular Vesicles

Endometriosis is defined as the presence of endometrial tissue outside the uterus, which troubles 25-50% women at their reproductive age (40, 41). While endometriosis is supposed to a benign inflammatory gynecological disease, some malignant biological behaviors (including invasion (42), recurrence and so on) also make it one of main reasons for infertility.

Recent studies showed that extracellular vesicles are associated with angiogenesis (43), cell proliferation, and gene mutation in endometriosis. Among these effects, different biological behaviors are mediated by different encapsulated content in extracellular vesicles. Previous studies confirmed that extracellular vesicles and/or extracellular vesicles-derived microRNA-126-5p (44) and proteins could regulate the proliferation, migration of endometrial mesenchymal stem cells by negatively regulating the expression of BCAR3 (a kind of EMT-associated genes), as well as enhance the angiogenic abilities, subsequently affect the occurrence and metastasis of endometriosis. Although BCAR3 was not associated with synergistic effect with estrogen and not associated with inducing EMT, its inhibition of anti-estrogen function may provide new insight into the mechanism of local estrogen action in endometriosis (45). Studies have shown that endometriosis stromal cells could enhance the angiogenic ability *in vitro* through secreted extracellular vesicles, and many other cell types also exert angiogenic effects through extracellular vesicles in regulating endothelial cells (46) and stromal cells (47). Based on the next-generation sequencing of EVs obtained from endometriosis patient plasma–derived extracellular vesicles compared with healthy control extracellular vesicles, studies have documented that differential expression of miR-16 and -30d regulating the angiogenic function by targeting the VEGF and MYPT1/cJUN/VEGFA pathway, respectively. These results suggesting that extracellular vesicles derived from endometriosis exert their contribution to the pathophysiology process of angiogenesis and invasion (43). Furthermore, the identification of biomarkers for the early diagnosis in endometriosis is essential to protect the gradual aggravation of the disease (48) (**Figure 1**).

### Polycystic Ovary Syndrome (PCOS) and Extracellular Vesicles

Polycystic Ovary Syndrome (PCOS), a kind of reproductive endocrine disorder which troubles women at childbearing age (49). PCOS is characterized by ovulation disorder, hyperandrogenism, and an excessive number of follicles (equal or greater than 12 follicles) of unilateral ovarian, is regarded as one of the most common causes of infertility. It is reported that the incidence increased for the reason that the transformation of the life-style and elevated related-risks [including obesity (50), insulin resistance (51) and so on] in recent years.

The existence of extracellular vesicles in human follicular fluid may provide pathways for information exchange between follicular fluid microenvironment and the oocyte (16, 52). The miRNAs in extracellular vesicles might play a regulatory role in the pathogenesis of PCOS (53, 54). Platelet-derived extracellular vesicles was detected elevated in plasma of women with PCOS when compared to healthy women. In addition, the extracellular vesicles derived from platelet are correlated with the serum testosterone levels (55), and similarly correlated with the free androgen index. Further studies reported that the extracellular vesicles derived from platelet are significantly elevated in obese women with PCOS, even overweight women with PCOS (56). Other study founded that PCOS women had higher concentrations of extracellular vesicles, further studies indicated that when focusing on the sub-population of small extracellular vesicles whose diameter less than 150 nm, small extracellular vesicles from PCOS women expressed greater percentage of annexin V positive than control women (56).

Recently study demonstrated that the results by miRNA profiling indicate that extracellular vesicles encapsulated hsa-miR-126-3p (53), ciRNA-7323_TIAM1 (57), circLDLR (58) have been altered in women with PCOS. And depleting circLDLR in extracellular vesicles would increase the expression level of miR-1294 and inhibit the expression level of CYP19A1 in recipient cells. In addition, down-regulated circLDLR in extracellular vesicles functioned as a vital mediator to regulate E2 secretion *via* sponging miR-1294 to repress CYP19A1 (58). Extracellular vesicles encapsulated miRNAs might exert potentially effects on the IGF1R signaling pathways upon the recipient cells in PCOS patients (59), which were different from the effects of non-extracellular vesicles-mediated miRNA secretion. These results would not only broaden the understanding of molecular mechanism in PCOS, but also provide new insights and strategies for further therapies against PCOS.

### Primary Ovary Insufficiency (POI) and Extracellular Vesicles

Primary Ovary Insufficiency (POI), a kind of disorder known as premature ovarian failure or premature menopause defined as cessation of menstruation before the expected age of menopause (60). While POI could be divided into genetic, autoimmune, and iatrogenic categories (61), evidences indicate that extracellular vesicles is related to the progression and treatment of POI. It was reported that the extracellular vesicles derived microRNAs is associated with POI. In addition, some studies reported that extracellular vesicles derived from human adipose mesenchymal stem cells would attenuate the ovary function damage through SMAD signaling pathway in a POI mouse model (62). Also, extracellular vesicles derived from human umbilical cord mesenchymal stem cells (hUMSCs) encapsulated miR-17-5P repressed PARP1, γH2AX, and XRCC6 by inhibiting SIRT7 (63), which implied the potential of extracellular vesicles based therapy for POI treatment. Extracellular vesicles derived bone mesenchymal stem cell (BMSC) transferred miR-644-5p could inhibit the apoptosis of ovarian granulosa cell by targeting p53 of cells (64), suggesting that the potential of extracellular vesicles as nano-carriers in treating POI as well as restoring ovarian function.

## Male Infertility and Extracellular Vesicles

It was reported that among all the couples suffered from infertility worldwide, 20-30% of them resulted from male infertility (65), while only 20-35% resulted from female infertility. However, male infertility (66) is often undervalued in the routine clinical practice.

Extracellular vesicles transferred proteins and miRNAs play a vital role in the multi-steps process including sperm motility (67), capacitation, acrosome reaction, and further fertilization. Studies demonstrated that extracellular vesicles proteins play role in the process of cell growth, cell maintenance and protein metabolism. Further results indicated that the extracellular vesicles proteome of normozoospermic men differs from non-normozoospermic men. Proteins known as positively regulators on sperm-specific functions including sperm associated antigen 11B (SPAG11B), cysteine-rich secretory protein-1 (CRISP1), and defensin B126 (DEFB126), were most strongly enriched in extracellular vesicles samples from seminal plasma of normozoospermic men; on the other hand, glycodelin (PAEP) and TGM4, were among the more represented proteins in extracellular vesicles from severe asthenozoospermic samples (68), suggesting that extracellular vesicles proteome might be potential biomarker in predicting the potential outcome (69). Aberrant expression of extracellular vesicles proteins could affect sperm functions and influence the subsequent fertilization. In mice, some studies demonstrated that the loss of specific proteins in extracellular vesicles causes infertility.

Besides proteins in extracellular vesicles, several studies have shown that aberrant miRNA levels in seminal plasma derived small extracellular vesicles (sEVs) are related to the sperm quality (70). Extracellular vesicles derived seminal plasma could potentially regulate the signaling pathways of the recipient mucosa through delivering the small RNA molecules. Some studies identified that when compared with controls, several in seminal plasma extracellular vesicles derived miRNAs altered in azoospermic individuals. It was reported that miR-31-5p in extracellular vesicles from semen would act as a predictive biomarker for the origin of azoospermia with high sensitivity and specificity, and the prediction efficacy was even better when combined the blood FSH values in the analysis (20). In addition, other studies demonstrated the biological role of extracellular vesicles beyond the epididymis and even outside the male reproductive tract (71). Extracellular vesicles from the ejaculates of normozoospermic men (including men after vasectomy) would significantly increase the sperm motility, while extracellular vesicles from asthenozoospermic men damage the sperm motility. Extracellular adenosine triphosphate produced in seminal plasma extracellular vesicles may finely modulate mitochondrial metabolism to control sperm motility (72). The results can provide insights into semen dilution and artificial insemination. Other studies reported that when spermatozoa isolated from two different severe asthenozoospermic patients coincubated with extracellular vesicles from seminal plasma of normozoospermic men, CRISP1 protein levels increased in spermatozoa treated with extracellular vesicles, as did those of lysosomal-associated membrane protein 1 (LAMP1), a canonic extracellular vesicles marker, strongly suggesting that extracellular vesicles-mediated transfer in regulating sperm motility (68). What’s more, better understanding of the spatiotemporal contents of extracellular vesicles and aberrant fluctuation of encapsulated component, and further the mechanism of regulation upon sperm will be critical to better understanding fertility and developing potential treatments in the future.

## Extracellular Vesicles and In-Vitro Fertilization (IVF)

The technology of in-vitro fertilization (IVF) has underwent rapid development since it came out (73). Although IVF technology is originally used for women with tubal factor infertility, it has been regarded as the last resort treatment of for all infertility couples when conventional therapy fails. However, how to better understand the biological process (including molecular regulation and environmental regulation) during the whole in-vitro fertilization, and how to improve IVF pregnancy rates still undiscovered.

Although it was well-acknowledged that technology of intracytoplasmic sperm injection has brought many successful pregnancies by evading the obstacle in conception (74) (including low sperm count and so on), the success rate of the technology still remains suboptimal. The increasing understanding of the role of extracellular vesicles in fertility process is vital in the assisted reproduction. It was demonstrated that the sperm RNAs involved in the regulation during the process of fertilization and further embryo development (5), and the extracellular vesicles microRNAs derived from human follicular fluid are involved in critically important pathways (including WNT, MAPK, ErbB, and TGFb signaling pathway) for follicle growth and oocyte maturation, which also explaining the correlation between the lack of extracellular vesicle–delivered RNAs and poorer outcomes among azoospermic men after successful microscopic testicular sperm extraction. Also, these results could represent noninvasive biomarkers of oocyte quality or sperm quality in assisted reproductive technology (75).

It is also reported that that the fallopian tube is superior for fertilization and embryo development than artificially modified conditions *in vitro*. Nevertheless, we still cannot pin-point which proteins or molecular cargos from extracellular vesicles are responsible for normal embryo development. It was reported that extracellular microRNAs in follicular fluid could lead to downstream events that would affect fertilization and embryo morphology (76). What’ more, some studies demonstrated that several key components derived from extracellular vesicle in the follicular microenvironment might be potential to act as predicting factors for the pregnancy outcomes in Assisted Reproductive Technology (ART) (40). These results indicate that extracellular vesicles might associated with fertilization potential and embryo quality. However, it is also still uncertain how extracellular vesicles regulate the optimal microenvironment for gametes and embryos in the multi-steps process in humans.

## Extracellular Vesicles as Potential Therapeutics in Fertility

Considering that extracellular vesicles are stable and low-immunogenicity, their therapeutic applications as drug delivery systems have drawn great attention in the treatment area. Combination of the complicated bio-engineering nanotechnology not only enable the encapsulation of therapeutic agents such as miRNAs and small molecules into extracellular vesicles, but also modify the extracellular vesicles with diverse ligands as targeting nano-carriers.

Increasing studies regarding the use of extracellular vesicles in the treatment of infertility was explored with respect to not only in female infertility (including PCOS, POI and endometriosis) but also male infertility. It was reported that extracellular vesicles encapsulated miR-214 could reduce the expression of Collagen αI and CTGF in endometriosis stromal and endometrial epithelial cells both *in vitro* and *in vivo*, and further alleviate the endometriosis fibrosis. Some studies demonstrated that mesenchymal stem cells derived extracellular vesicles could promote proliferation and inhibits apoptosis of cumulus cells in polycystic ovary syndrome (PCOS) *via* transferring encapsulated miR-323-3p and targeting PDCD4. And upregulation of miR-323-3p ameliorated PCOS *via* regulating the serum FSH, LH and E2 levels in the PCOS mice model (77). Recent study demonstrated that histopathological evaluation provided evidences that spermatogenesis would be improved when treated with extracellular vesicles derived from amniotic fluid in non-obstructive azoospermia rats through injection treatment, which indicate that extracellular vesicles are potential to orchestrate the sperm quality and further recovery of sperm production capacity.

The above results suggest that therapeutic extracellular vesicles can be explored and applied in infertility. Although most of studies remain in the in-vitro and animal level, and challenges for clinical application still unsolved, the drug delivery based on engineering extracellular vesicles still remains a promising therapeutic strategy.

## Summary and Perspectives

As a kind of disorder which disturbs numerous couples at reproductive age, infertility has drawn widespread attentions for the reason that the rapidly increasing among generations. In spite of the understanding of infertility as well as the rapid development of Assisted Reproductive Technology(ART), some limitations including unsatisfactory rate of success, undiscovered mechanism and limited therapeutics still remain.

While increasing studies demonstrate the correlation between the extracellular vesicles (including concentration, size and specific cargos) and infertility, the underlying mechanism of extracellular vesicles function in the process of infertility is still unclear. In addition, most of the current studies of extracellular vesicles in reproduction and infertility still remains the animal models, more relevant human-related research is needed. Taken together, extracellular vesicles play an important role in mediating a variety of physiological and pathological processes through the intracellular communication and exchange of substance, which provides us a promising avenue to better understand and subsequent treat infertility (23).

## Author Contributions

GZho: writing-original draft and editing. YG: writing-original draft. FZ: writing-review and editing, visualization. MZ: writing-review and editing. GZha: writing-review and editing, LW: review and editing. KH: review and editing. JD: writing-review and editing, supervision, and funding acquisition. All authors contributed to the article and approved the submitted version.

## Funding

This work was supported by the National Natural Science Foundation of China (No. 81771524), National Natural Science Foundation of China (No. 91440107).

## Conflict of Interest

The authors declare that the research was conducted in the absence of any commercial or financial relationships that could be construed as a potential conflict of interest.

## Publisher’s Note

All claims expressed in this article are solely those of the authors and do not necessarily represent those of their affiliated organizations, or those of the publisher, the editors and the reviewers. Any product that may be evaluated in this article, or claim that may be made by its manufacturer, is not guaranteed or endorsed by the publisher.
